# A Dictionary of Medical Science

**Published:** 1858-04

**Authors:** 


*A Dictionary af Medical Science:* containing a concise Explanation of the various Subjects and Items of Anatomy, Physiology, Pathology, Hygiene, Therapeutics, Pharmacology, Pharmacy, Surgery, Obstetrics, Medical Jurisprudence, Dentistry, &c.; Notices of Mineral Waters, and of Climate; Formulæ for Officinal, Empirical and Dietetic Preparations, &c.; with French and other Synonymes. By Robley Dunglison, M. D., LL.D.—Philadelphia: Blanchard & Lea, 1857.

Such is the prolonged title of one of the most laborious compilations ever issued by the press. The great value of the book has been long known and universally acknowledged. Its copious learning, its general accuracy, its extreme minuteness have made it a favorite every where. So thoroughly is every one who reads medicine acquainted with it, that any commendations of it at this late day are useless.

All that is necessary for us to say is, that the author's industry has made this revised addition still more worthy of professional attention. About six thousand new terms have been added, and the work has been greatly enlarged.

